# Supplementary material for: The Alpha Subunit of Mitochondrial Processing Peptidase Participated in Fertility Restoration in Honglian-CMS Rice
Source: Int J Mol Sci. 2023 Mar 13;24(6):5442. doi: 10.3390/ijms24065442 (PMC10049570; doi:10.3390/ijms24065442)
Supplement: Supplementary file 1 [file ijms-24-05442-s001.zip › Supplementary figures.pptx]

## Slide 1
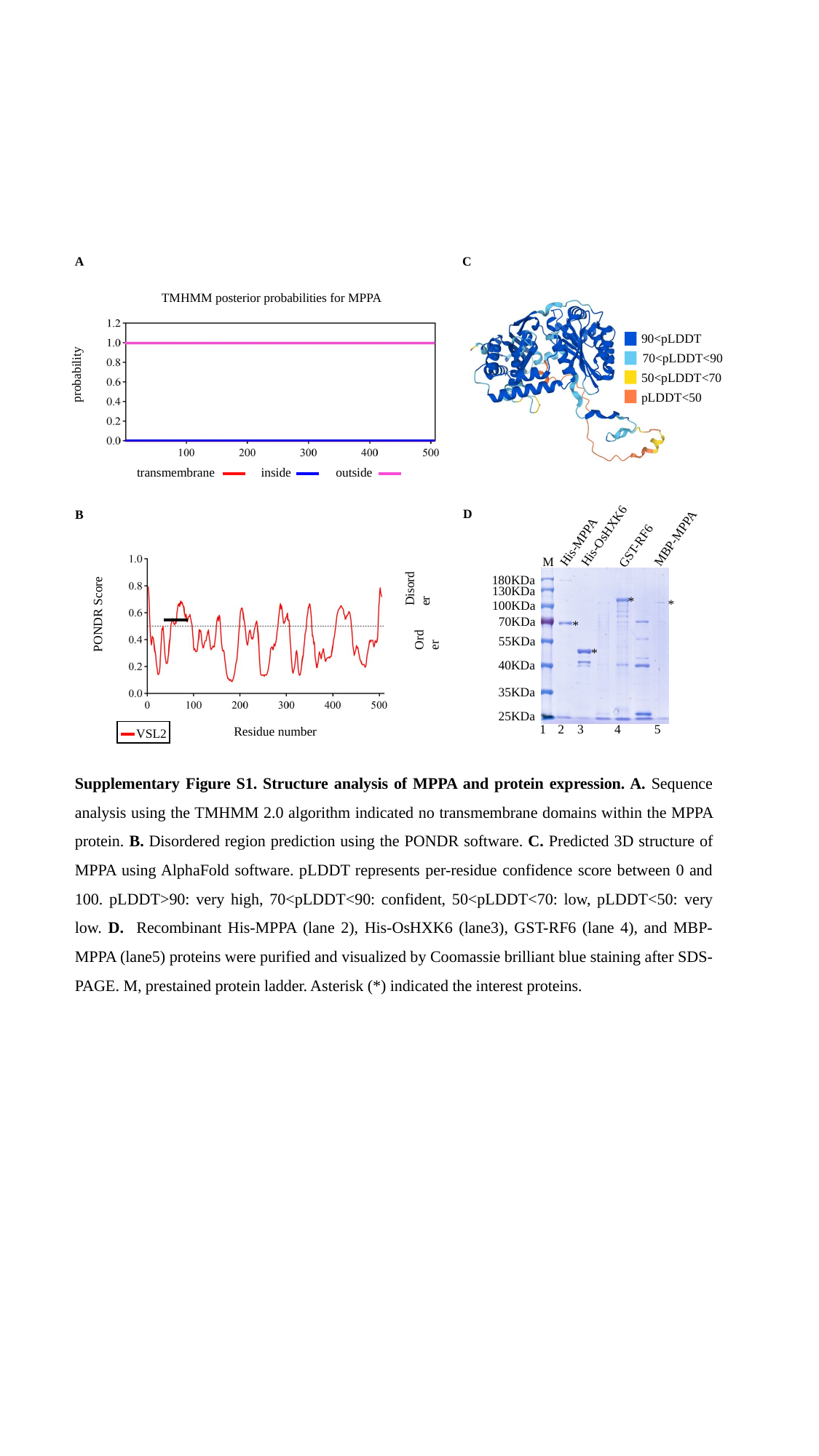

C
A
TMHMM posterior probabilities for MPPA
probability
inside
outside
transmembrane
90<pLDDT
70<pLDDT<90
50<pLDDT<70
pLDDT<50
His-OsHXK6
MBP-MPPA
His-MPPA
GST-RF6
M
180KDa
130KDa
100KDa
70KDa
55KDa
40KDa
35KDa
25KDa
1
2
3
4
5
*
*
*
*
D
B
PONDR Score
Disorder
Order
Residue number
VSL2
Supplementary Figure S1. Structure analysis of MPPA and protein expression. A. Sequence analysis using the TMHMM 2.0 algorithm indicated no transmembrane domains within the MPPA protein. B. Disordered region prediction using the PONDR software. C. Predicted 3D structure of MPPA using AlphaFold software. pLDDT represents per-residue confidence score between 0 and 100. pLDDT>90: very high, 70<pLDDT<90: confident, 50<pLDDT<70: low, pLDDT<50: very low. D. Recombinant His-MPPA (lane 2), His-OsHXK6 (lane3), GST-RF6 (lane 4), and MBP-MPPA (lane5) proteins were purified and visualized by Coomassie brilliant blue staining after SDS-PAGE. M, prestained protein ladder. Asterisk (*) indicated the interest proteins.

## Slide 2
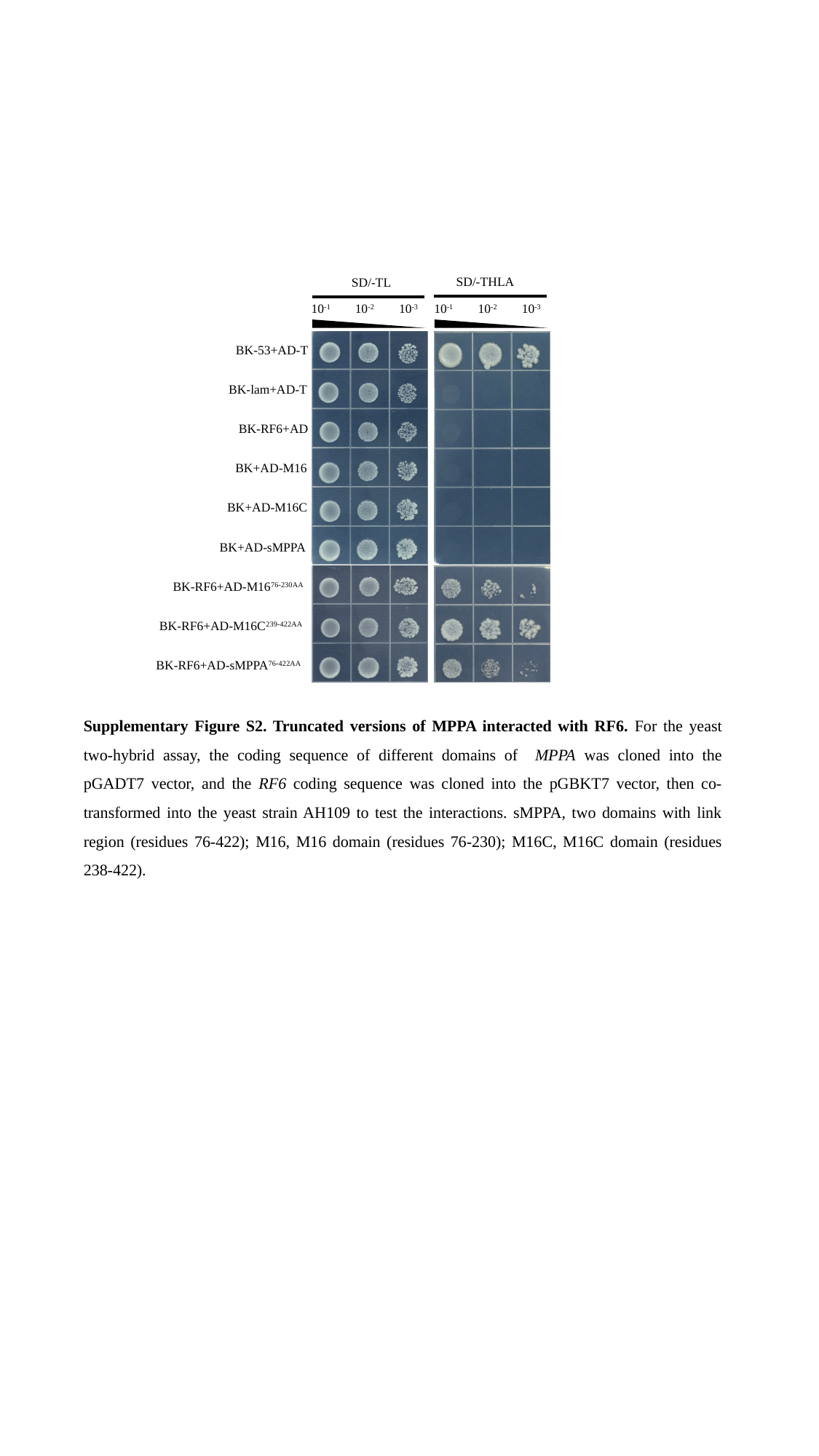

SD/-THLA
10-1
10-2
10-3
SD/-TL
10-1
10-2
10-3
BK-53+AD-T
BK-lam+AD-T
BK-RF6+AD
BK+AD-M16
BK+AD-M16C
BK+AD-sMPPA
BK-RF6+AD-M1676-230AA
BK-RF6+AD-M16C239-422AA
BK-RF6+AD-sMPPA76-422AA
Supplementary Figure S2. Truncated versions of MPPA interacted with RF6. For the yeast two-hybrid assay, the coding sequence of different domains of MPPA was cloned into the pGADT7 vector, and the RF6 coding sequence was cloned into the pGBKT7 vector, then co-transformed into the yeast strain AH109 to test the interactions. sMPPA, two domains with link region (residues 76-422); M16, M16 domain (residues 76-230); M16C, M16C domain (residues 238-422).

## Slide 3
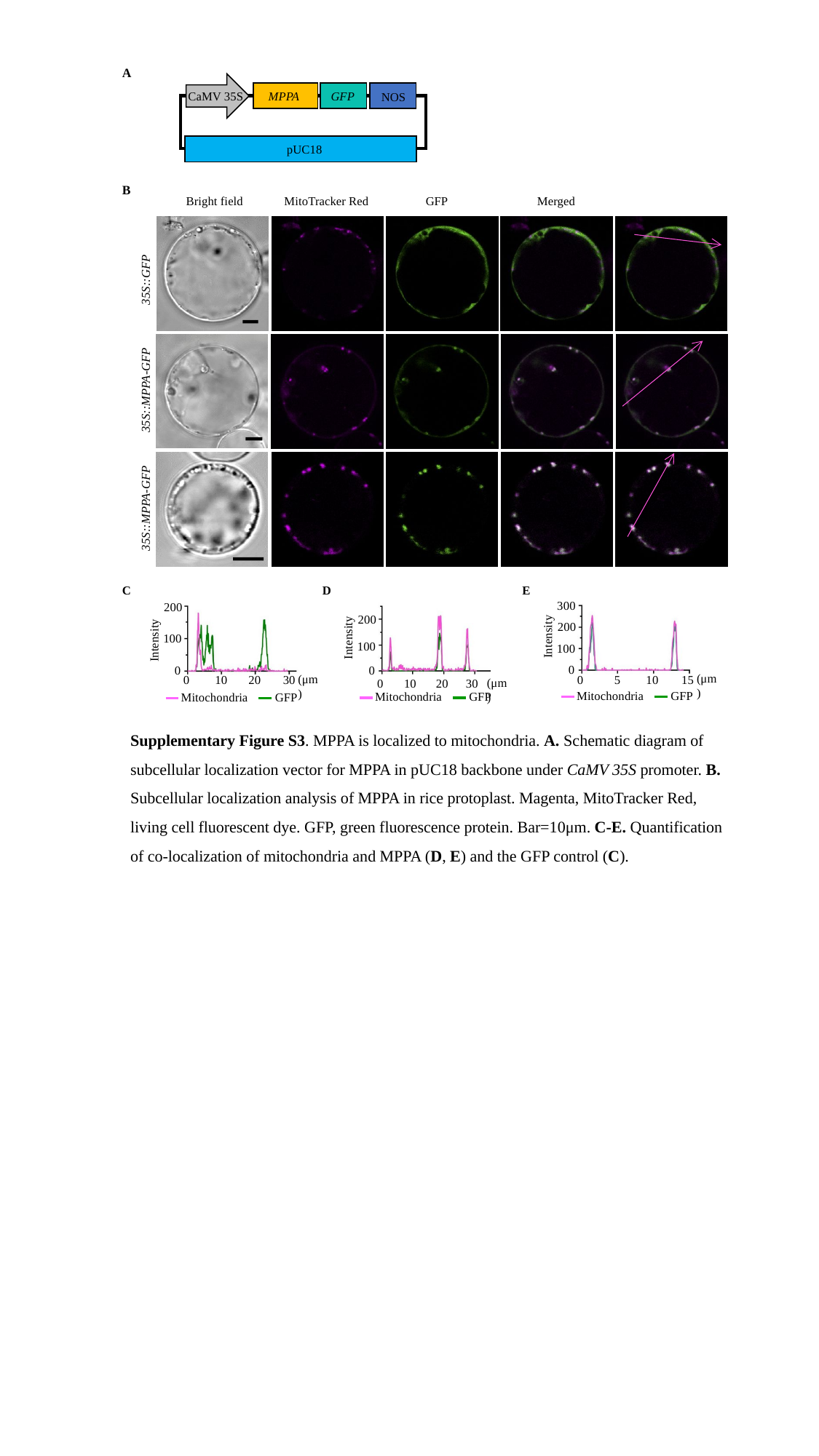

A
CaMV 35S
MPPA
NOS
GFP
pUC18
B
Bright field
MitoTracker Red
GFP
Merged
35S::GFP
35S::MPPA-GFP
35S::MPPA-GFP
C
D
E
300
200
Intensity
100
0
(μm)
0
5
10
15
Mitochondria
GFP
200
100
Intensity
0
(μm)
0
10
20
30
Mitochondria
GFP
200
Intensity
100
0
(μm)
0
10
20
30
Mitochondria
GFP
Supplementary Figure S3. MPPA is localized to mitochondria. A. Schematic diagram of subcellular localization vector for MPPA in pUC18 backbone under CaMV 35S promoter. B. Subcellular localization analysis of MPPA in rice protoplast. Magenta, MitoTracker Red, living cell fluorescent dye. GFP, green fluorescence protein. Bar=10μm. C-E. Quantification of co-localization of mitochondria and MPPA (D, E) and the GFP control (C).

## Slide 4
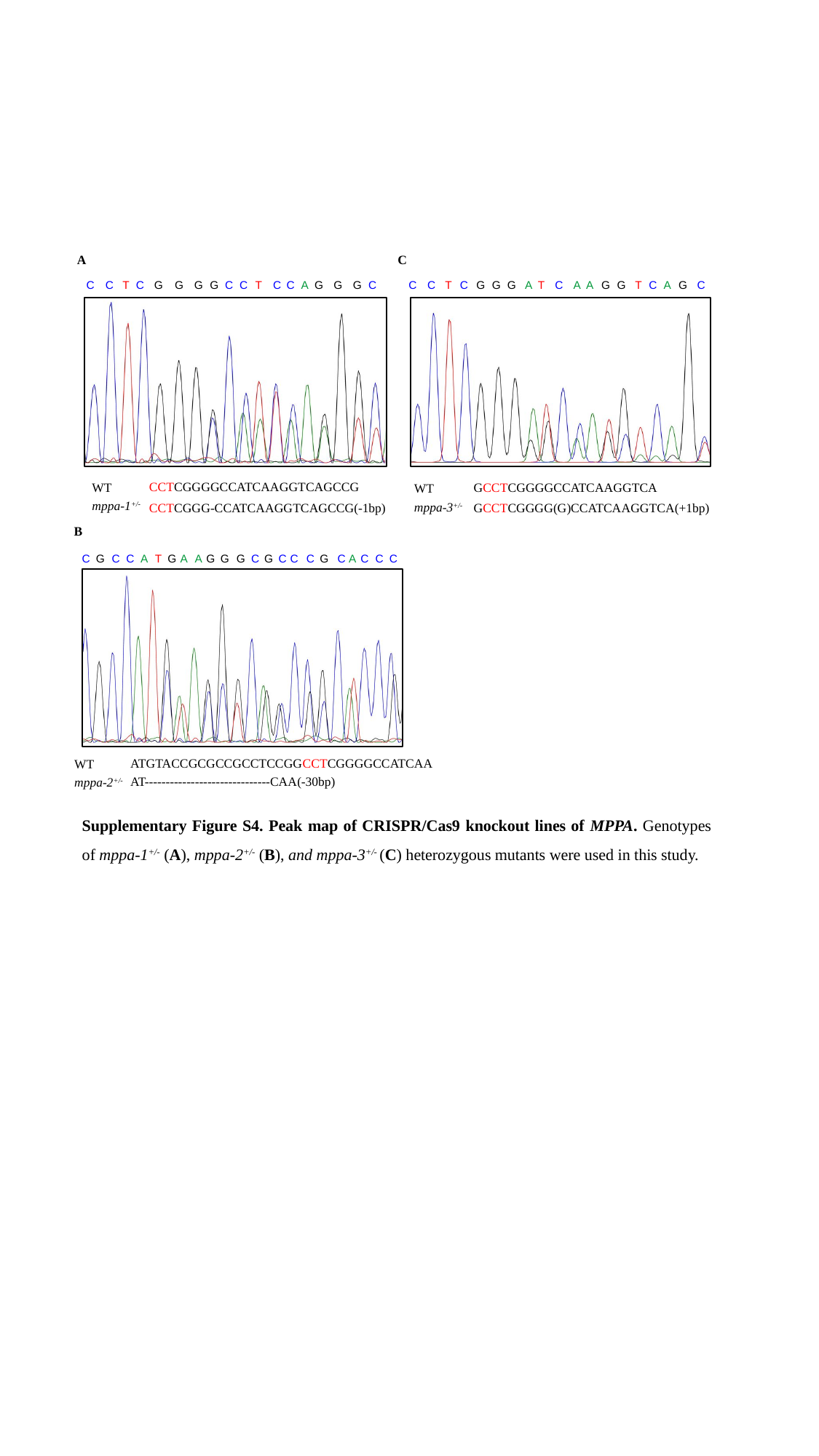

A
C
C
C
T
C
G
G
G
G
C
C
T
C
C
A
G
G
G
C
CCTCGGGGCCATCAAGGTCAGCCG
WT
mppa-1+/-
CCTCGGG-CCATCAAGGTCAGCCG(-1bp)
C
C
T
C
G
G
G
A
T
C
A
A
G
G
T
C
A
G
C
GCCTCGGGGCCATCAAGGTCA
WT
mppa-3+/-
GCCTCGGGG(G)CCATCAAGGTCA(+1bp)
B
C
G
C
C
A
T
G
A
A
G
G
G
C
G
C
C
C
G
C
A
C
C
C
ATGTACCGCGCCGCCTCCGGCCTCGGGGCCATCAA
WT
AT------------------------------CAA(-30bp)
mppa-2+/-
Supplementary Figure S4. Peak map of CRISPR/Cas9 knockout lines of MPPA. Genotypes of mppa-1+/- (A), mppa-2+/- (B), and mppa-3+/- (C) heterozygous mutants were used in this study.

## Slide 5
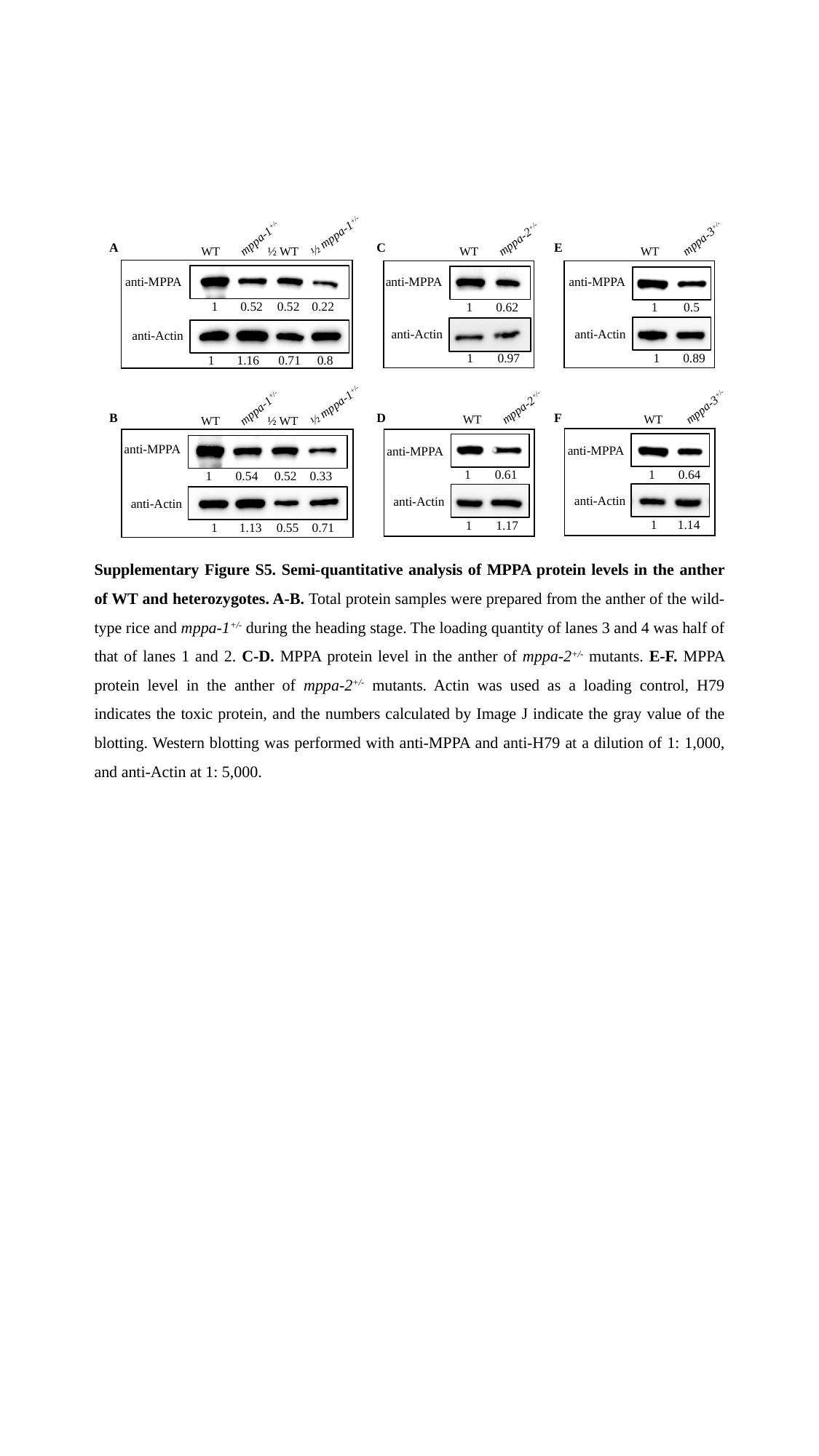

½ mppa-1+/-
mppa-3+/-
mppa-1+/-
mppa-2+/-
A
C
E
WT
½ WT
WT
WT
1
0.52
0.52
0.22
anti-MPPA
1
1.16
0.71
0.8
anti-Actin
1
0.62
1
0.97
anti-MPPA
anti-Actin
1
0.5
1
0.89
anti-MPPA
anti-Actin
½ mppa-1+/-
mppa-3+/-
mppa-2+/-
mppa-1+/-
B
D
F
WT
WT
WT
½ WT
1
0.64
1
1.14
anti-MPPA
anti-Actin
1
0.61
1
1.17
anti-MPPA
anti-Actin
anti-MPPA
1
0.54
0.52
0.33
1
1.13
0.55
0.71
anti-Actin
Supplementary Figure S5. Semi-quantitative analysis of MPPA protein levels in the anther of WT and heterozygotes. A-B. Total protein samples were prepared from the anther of the wild-type rice and mppa-1+/- during the heading stage. The loading quantity of lanes 3 and 4 was half of that of lanes 1 and 2. C-D. MPPA protein level in the anther of mppa-2+/- mutants. E-F. MPPA protein level in the anther of mppa-2+/- mutants. Actin was used as a loading control, H79 indicates the toxic protein, and the numbers calculated by Image J indicate the gray value of the blotting. Western blotting was performed with anti-MPPA and anti-H79 at a dilution of 1: 1,000, and anti-Actin at 1: 5,000.

## Slide 6
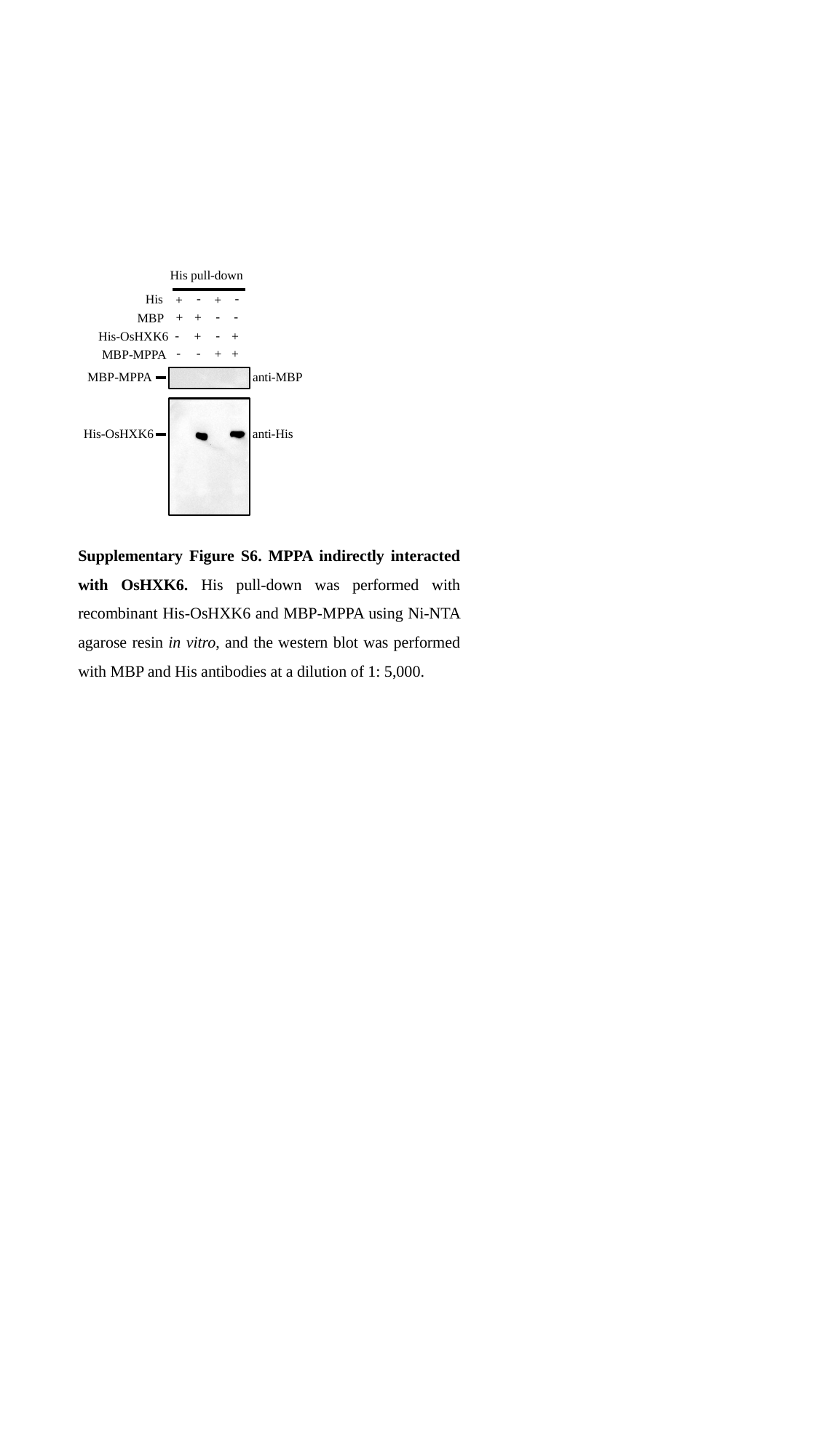

His pull-down
-
-
His
+
+
-
-
+
+
MBP
-
-
+
+
His-OsHXK6
-
-
+
+
MBP-MPPA
MBP-MPPA
anti-MBP
anti-His
His-OsHXK6
Supplementary Figure S6. MPPA indirectly interacted with OsHXK6. His pull-down was performed with recombinant His-OsHXK6 and MBP-MPPA using Ni-NTA agarose resin in vitro, and the western blot was performed with MBP and His antibodies at a dilution of 1: 5,000.

## Slide 7
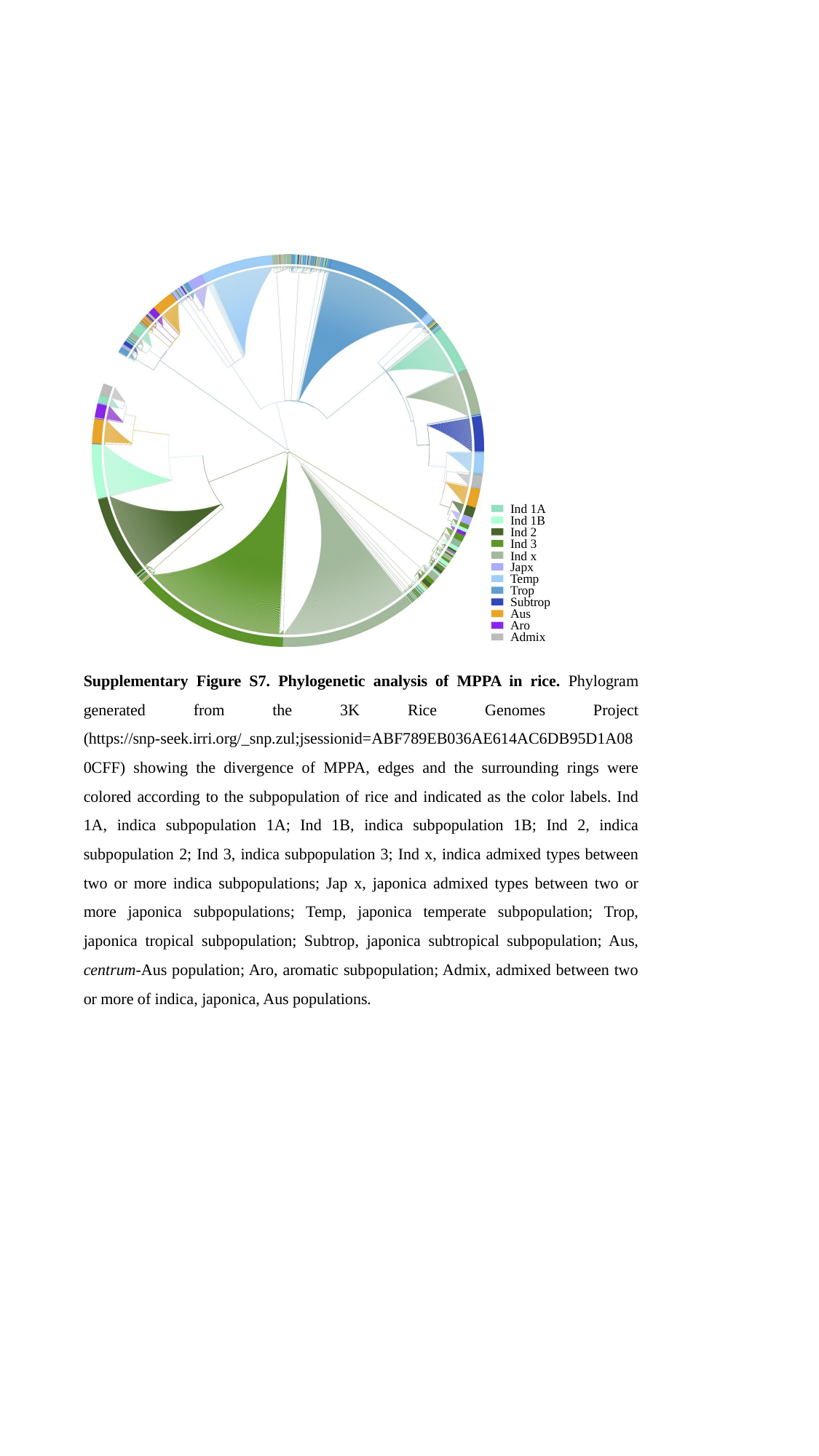

Ind 1A
Ind 1B
Ind 2
Ind 3
Ind x
Japx
Temp
Trop
Subtrop
Aus
Aro
Admix
Supplementary Figure S7. Phylogenetic analysis of MPPA in rice. Phylogram generated from the 3K Rice Genomes Project (https://snp-seek.irri.org/_snp.zul;jsessionid=ABF789EB036AE614AC6DB95D1A080CFF) showing the divergence of MPPA, edges and the surrounding rings were colored according to the subpopulation of rice and indicated as the color labels. Ind 1A, indica subpopulation 1A; Ind 1B, indica subpopulation 1B; Ind 2, indica subpopulation 2; Ind 3, indica subpopulation 3; Ind x, indica admixed types between two or more indica subpopulations; Jap x, japonica admixed types between two or more japonica subpopulations; Temp, japonica temperate subpopulation; Trop, japonica tropical subpopulation; Subtrop, japonica subtropical subpopulation; Aus, centrum-Aus population; Aro, aromatic subpopulation; Admix, admixed between two or more of indica, japonica, Aus populations.
